# A Case of Euglycemic Ketoacidosis Secondary to Continuous Renal Replacement Therapy

**DOI:** 10.1155/crcc/6275218

**Published:** 2025-01-03

**Authors:** Evan J. Chen, William A. West, Kavitha Bagavathy

**Affiliations:** ^1^Department of Pulmonary & Critical Care Medicine, Los Angeles General Medical Center, Los Angeles, California, USA; ^2^Division of Pulmonary, Critical Care & Sleep Medicine, Keck Hospital of USC, Los Angeles, California, USA

**Keywords:** anion gap metabolic acidosis, beta-hydroxybutyrate, continuous renal replacement therapy, enteral feeding, euglycemic diabetic ketoacidosis, euglycemic ketoacidosis, insulin

## Abstract

Euglycemic ketoacidosis (EKA) has been reported as a rare but life-threatening complication of continuous renal replacement therapy (CRRT). EKA should be suspected in the setting of persistent high anion gap metabolic acidosis despite renal replacement therapy. Critically ill patients, especially those with diabetes mellitus, are at risk of EKA due to deficient caloric intake, the presence of excess counterregulatory stress hormones, and nutritional losses from CRRT. Even with the use of glucose-containing dialysates, EKA can be observed. Prompt treatment with insulin and glucose-containing infusions leads to rapid resolution of the condition. Early optimization of nutritional intake can prevent or mitigate EKA. This case report describes a patient who developed EKA while on CRRT for severe acute kidney injury from neuroleptic malignant syndrome.

## 1. Introduction

Euglycemic ketoacidosis (EKA) has been reported as a rare but life-threatening complication of continuous renal replacement therapy (CRRT). EKA is diagnosed by the presence of ketonemia, high anion gap metabolic acidosis (HAGMA) (with serum bicarbonate < 18 meq/L and/or pH < 7.30), and euglycemia (serum glucose < 250 mg/dL) [1, 2]. EKA is usually caused by decreased caloric intake or starvation, excessive alcohol intake, pregnancy, chronic liver disease, pancreatitis, and the use of sodium–glucose cotransporter-2 inhibitors [3–6]. Deficient caloric intake and nutritional losses from CRRT can also predispose critically ill patients to EKA. This case report describes a patient who developed EKA while on CRRT for severe acute kidney injury from neuroleptic malignant syndrome.

## 2. Case Presentation

**Figure 1:**
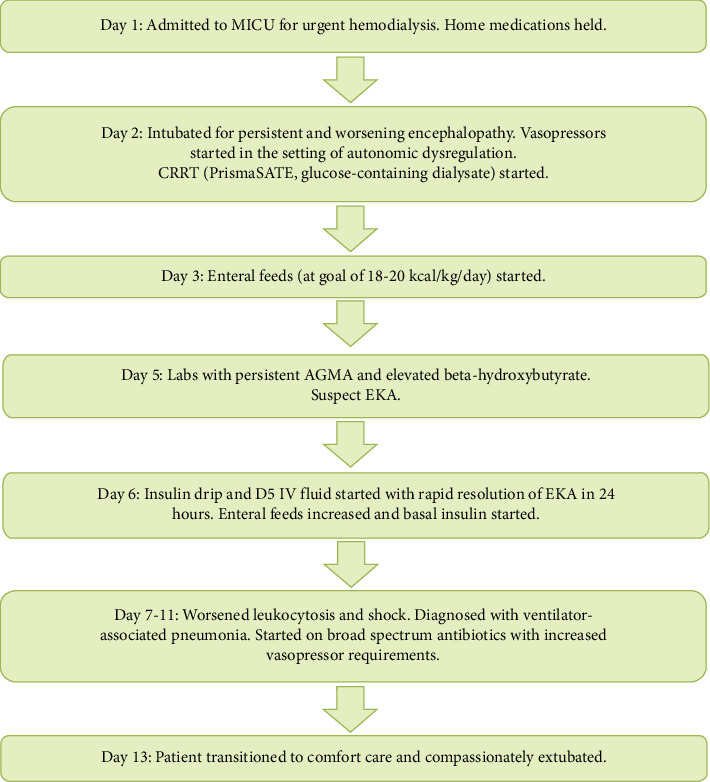
Summary of the patient's hospital course.

A 59-year-old male with a past medical history of Type 2 diabetes and schizophrenia presented to the emergency department with acute encephalopathy. Home medications included haloperidol and citalopram. He denied recent use of sodium–glucose cotransporter-2 inhibitors or recreational drug use. Vital signs on presentation were notable for tachycardia and hyperthermia with a temperature of 42°C. Physical exam was significant for diffuse muscle rigidity. Labs were notable for acute kidney injury, rhabdomyolysis, HAGMA, lactic acidosis, and unremarkable toxic alcohol and urine toxicology screens. A diagnosis of neuroleptic malignant syndrome was made. All antipsychotics were discontinued. He was started on empiric antibiotics for possible sepsis. Given worsening HAGMA, intermittent hemodialysis was initiated. However, he later developed hemodynamic instability requiring vasopressor support and worsening mental status requiring intubation. Intermittent hemodialysis was transitioned to CRRT (PrismaSATE, glucose-containing dialysate). Enteral feeds were also started (at a goal of 18–20 kcal/kg/day). Despite improvement in lactic acidosis and uremia, a persistent HAGMA was observed (Table 1). There was no concern for citrate toxicity or use of medications known to cause HAGMA. Further studies revealed an elevated serum beta-hydroxybutyrate level (Table 1). In the setting of euglycemia, the patient was suspected to have a diagnosis of EKA. He was started on insulin and dextrose-containing fluid infusions with resolution of HAGMA within 24 h (Table 1). Enteral feeds were subsequently increased (to a goal of 20–23 kcal/kg/day), and basal insulin was started. Unfortunately, due to his worsening clinical status from ventilator-associated pneumonia and septic shock, he was eventually transitioned to comfort care (Figure 1).

## 3. Discussion

Critically ill patients are at risk for developing HAGMA due to the presence of lactic acidosis, uremia, or medication use, all of which may resolve with initiation of CRRT and discontinuation of the offending medication. However, EKA should be suspected in the setting of persistent HAGMA despite CRRT. EKA occurs due to relative or absolute carbohydrate deficit and insulin resistance or deficiency, leading to increased glucagon-to-insulin ratio. This leads to the deprivation of intracellular glucose stores, lipolysis, and production of ketoacids [5]. Critically ill patients, especially those with diabetes mellitus, are at risk of EKA due to deficient caloric intake, the presence of excess counterregulatory stress hormones, and nutritional losses from CRRT [3, 4, 7]. The use of glucose-free dialysates during CRRT could also contribute to EKA, as approximately 40–80 g/day of glucose is lost during CRRT, thereby furthering the negative caloric balance in critically ill patients [8]. However, few studies, as seen in our patient's case, showed that EKA can be observed even with the use of glucose-containing dialysates [4, 7, 9]. This underscores the need for optimizing a patient's caloric intake as early as tolerated. Our patient's case had many features consistent with previous reports of CRRT-associated EKA but also had features not previously described; namely, our patient developed persistent HAGMA despite having a normal serum pH at diagnosis, which was likely due to CRRT and mechanical ventilation; this could have prevented further investigation into his underlying etiology of HAGMA.

Treatment of EKA involves increasing a patient's caloric intake to 25–35 kcal/kg/day, initiation of dextrose-containing fluids and insulin infusion, and often the addition of glucose in the dialysate [1, 3, 10]. Although limited by hemodynamic instability, early enteral feeds with optimal caloric intake should be achieved as early as tolerated. With treatment, EKA rapidly resolves within 24 h and is defined by normalization of the anion gap and improvement in acidosis (with serum bicarbonate > 18 meq/L and pH > 7.30) [1, 3, 4, 9].

This case highlights the importance of recognizing EKA as a complication of CRRT in patients with persistent HAGMA and the importance of early, optimal enteral feeding in critically ill patients. EKA is a life-threatening clinical condition that must be recognized early. Prompt treatment with insulin and glucose-containing infusions leads to its rapid resolution. Early enteral feeding and optimization of caloric replacements are crucial in preventing or mitigating EKA.

## Figures and Tables

**Table 1:** Laboratory trends prior to initiation of and during CRRT.

**Laboratory finding**	**On admission**	**Prior to CRRT**	**24 h after CRRT**	**At diagnosis of EKA**	**At resolution of IKEA**
pH	7.28	7.14	7.38	7.41	7.43
Sodium (mmol/L)	137	139	139	138	137
Potassium (mmol/L)	4.2	5.0	4.3	4.5	4.1
Chloride (mmol/L)	108	98	104	103	105
Bicarbonate (mmol/L)	10	10	13	18	24
Anion gap (mmol/L)	19	31	22	17	8
Blood urea nitrogen (mg/dL)	44	68	44	30	21
Creatinine (mg/dL)	2.23	4.28	2.96	1.97	1.70
Albumin (g/dL)	2.8	2.9	2.9	2.7	2.6
Beta-hydroxybutyrate (mmol/L)				2.16	
Glucose (mg/dL)	207	299	181	233	182
Lactate (mmol/L)	6.9	1.4	1.4	2.1	1.9
Hemoglobin A1c (%)	7.1				

## Data Availability

Data sharing is not applicable to this article as no new data were created or analyzed in this study.
